# Stress fracture and osteomyelitis in a patient with systemic lupus erythematosus

**DOI:** 10.1590/0100-3984.2017.0035

**Published:** 2018

**Authors:** Clarissa Canella, Flavia Costa, Adriana Danowisk, Alessandro Severo Alves de Melo, Edson Marchiori

**Affiliations:** 1 Clínica de Diagnóstico Por Imagem (CDPI), Rio de Janeiro, RJ, e Universidade Federal Fluminense (UFF), Niterói, RJ, Brazil; 2 Clínica de Diagnóstico Por Imagem (CDPI), Rio de Janeiro, RJ, Brazil; 3 Hospital dos Servidores do Estado, Rio de Janeiro, RJ, Brazil; 4 Universidade Federal Fluminense (UFF), Niterói, RJ, Brazil; 5 Universidade Federal do Rio de Janeiro (UFRJ), Rio de Janeiro, RJ, Brazil

**Figure 1:**
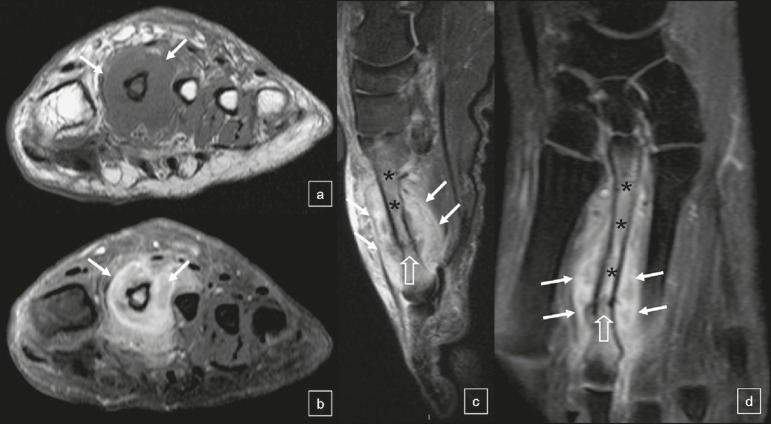
MRI of the left foot. Gadolinium contrast-enhanced axial T1-weighted MRI sequence (**a**), together with axial, sagittal, and coronal T1-weighted MRI sequences with fat suppression (**b, c,** and **d**, respectively), showing a diaphyseal fracture of the second metatarsal (open arrows) and extensive fluid collection with peripheral contrast enhancement of the surrounding tissue, indicating an abscess (arrows). Note the bone marrow enhancement, suggestive of osteomyelitis, in the second metatarsal (asterisks).

Dear Editor,

A 38-year-old woman who had been diagnosed with severe systemic lupus erythematosus (SLE) 15 years prior, had refractory nephritis, and had been treated with high-dose corticosteroids and immunosuppressive drugs (cyclophosphamide, mycophenolate mofetil, and rituximab), presented with a severalweek history of pain and edema on the dorsum of the left foot after wearing tight shoes. She had extremely low bone density, which had been treated with bisphosphonate and teriparatide. Magnetic resonance imaging (MRI) of the left foot showed a diaphyseal fracture of the second metatarsal with extensive fluid collection and peripheral contrast enhancement of the surrounding tissue, indicating an abscess (Figure 1). In addition, bone marrow edema of the second metatarsal with gadolinium enhancement suggested osteomyelitis.

Stress fractures may occur in SLE patients treated with corticosteroids, most commonly in the femoral head but also in the foot^(1)^. Atraumatic metatarsal stress fractures typically occur in association with antiphospholipid syndrome. Although the pathogenesis remains uncertain, it likely involves high bone strain and repetitive submaximal stress, causing microfractures and microinfarcts^(2-5)^. Other possible contributory factors include vasculopathy of the vessels supplying the bones and osteoporosis. Osteoporosis is usually observed in SLE patients, increasing the risk of fractures, and its pathogenesis is multifactorial. High disease activity and immobility are also common factors that substantially increase fracture risk in these patients, as do other factors such as age, body mass index, and gender.

Complications of stress fractures, including osteonecrosis, septic arthritis, and osteomyelitis, have also been described and can be associated with SLE. Patients with SLE are more prone to bacterial infection due to factors such as quantitative or qualitative deficiencies of complement proteins, renal dysfunction, impaired phagocytosis, impaired chemotaxis, and the use of immunosuppressants. Apart from appropriate imaging studies, patients suspected of having osteomyelitis should always undergo a complete sepsis workup, including blood, urine, and stool cultures. *Staphylococcus aureus* infection and opportunistic infections such as those caused by *Salmonella* spp. should be considered, as should tuberculosis in regions where it is prevalent. In the majority of cases, osteonecrosis is asymptomatic and occurs early in the course of the disease^(4)^. MRI is an excellent method for evaluating and diagnosing musculoskeletal disorders^(6-10)^, particularly complications related to bone fractures and osteonecrosis, thus allowing major sequelae to be avoided^(2-5)^. T1-weighted sequences with fat suppression after gadolinium administration, demonstrating soft tissue fluid collection with peripheral contrast enhancement, are essential for the diagnosis of abscess and osteomyelitis.

Treatment usually involves proper antimicrobial therapy, immobilization, and appropriate surgical treatment of infectious complications. For silent osteonecrosis involving a small area, conservative treatment is usually adequate. However, for lesions that are symptomatic, surgical interventions (core decompression or free vascularized bone grafting) are required. When the lesion involves a weight-bearing area, there can be bone collapse, which requires total joint replacement.
